# Sensor-Driven Deep Learning for Smart Home Intelligence: Signal Analysis, Multimodal Perception, and System-Level Applications

**DOI:** 10.3390/s26102993

**Published:** 2026-05-09

**Authors:** Chenchen Wu, Ziqian Yang, Tao Sun

**Affiliations:** 1 College of Furnishings and Industrial Design, Nanjing Forestry University, Nanjing 210037, China; 2 Nanjing Institute of Agricultural Mechanization, Ministry of Agriculture and Rural Affairs, Nanjing 210049, China

**Keywords:** smart homes, deep learning, human activity recognition, multimodal sensing, edge intelligence, sensor signal analysis, explainable AI, autonomous systems

## Abstract

Smart home environments are evolving toward context-aware intelligent systems with the rapid integration of the Internet of Things (IoT), edge computing, and artificial intelligence. In such settings, large volumes of heterogeneous sensor data must be continuously processed to support perception, behavior understanding, and autonomous decision-making. Deep learning has emerged as a key approach for transforming raw sensor signals into structured representations that enable these functions. This review examines recent advances in deep learning for smart home applications from a sensor-driven perspective. Existing studies are organized into five major domains: human activity recognition, health monitoring and assisted living, smart energy management, security monitoring and anomaly detection, and voice interaction and intelligent control. Representative methodological paradigms—including convolutional and recurrent neural networks, Transformers, graph-based learning, multimodal fusion, and deep reinforcement learning—are discussed with emphasis on their roles in signal representation, multimodal integration, and decision-oriented modeling. Despite notable progress, several challenges continue to limit real-world deployment. These include the scarcity of high-quality labeled data, privacy and security concerns associated with continuous sensing, limited generalization across environments and users, constraints of edge devices, and the limited interpretability of model output. Addressing these issues requires advances not only in model design but also in data-efficient learning, privacy-preserving architectures, and system-level integration. Future research is expected to focus on multimodal perception, distributed and edge intelligence, knowledge-enhanced modeling, and human-centered explainable systems. By synthesizing current developments and highlighting open challenges, this review aims to support the development of robust and deployable deep learning solutions for next-generation smart home systems.

## 1. Introduction

Driven by the development of the Internet of Things (IoT), sensor networks, cloud computing, and edge computing, people’s living environment is gradually evolving from a passive living environment to an intelligent system with the capability of perception, interconnection, computation, and autonomy. Backed by the infrastructure of ambient sensors, wearable gadgets, smart terminals, and home gateways, smart homes facilitate integrated management of illumination, security, thermal conditions, energy consumption, health care, and human–machine interaction to improve the quality of life, safety, and resource utilization [1]. Meanwhile, the population aging trend, increasing requirement for home healthcare, and awareness of energy efficiency are changing the research focus from device connectivity and rules-based automation to context-aware intelligence and customized services [1,2].

Technically, the first smart homes were based on rules and thresholds of activation, as well as on traditional machine learning algorithms. These techniques have been useful in a limited context but fail to provide flexibility and to handle the diversity and variability of real-world environments, especially in terms of feature engineering, model generalization, and cross-context adaptability [2]. Deep learning can learn hierarchical representations from sensor data and substantially reduce the reliance on manual feature engineering, although appropriate preprocessing steps, such as noise reduction, missing-value handling, normalization, and temporal alignment, remain necessary before model training and inference in smart home sensing applications [3]. The multimodal nature of smart home data, its temporal dependence, and contextual sensitivity are some of the features that have been exploited by deep learning approaches, making it a key enabler in this emerging technological field.

Human activity recognition (HAR) plays a critical role in this environment as it connects low-level sensing to high-level decision-making. The ability to identify user activities is reliable and gives the necessary behavioral context to applications such as anomaly detection, health assistance, adaptive control, and personalized services. Consequently, HAR has turned out to be one of the most researched areas in smart homes with the support of popular datasets and experimental platforms. The examples are representative, such as the CASAS platform used to monitor long-term behavior [1] or the UCI HAR dataset used to recognize activities based on wearable devices [4]. Based on these resources, convolutional and recurrent neural networks, hybrid architectures have been continuously enhanced in terms of recognition accuracy and robustness [5]. In this respect, HAR is not only a fundamental application area but also an important interface between perception, understanding and intelligent response.

Meanwhile, the usage of deep learning has not only been applied to activity recognition. In health surveillance, behavioral and environmental sensors are used to detect falls, sleep, and chronic illness, and these systems can be deployed in home environments [6]. Multimodal data is applied to security monitoring to identify intrusions, abnormalities, and safety threats, as well as for real-time detection [7]. Further progress in speech recognition and natural language processing has also contributed to voice-based interaction, enabling a more human-like and contextual control over devices [3]. All these changes have been seen as a general shift towards systems capable of understanding and adapting their services.

An analogous transition can be observed in smart energy management, where the penetration of distributed energy resources such as photovoltaics, energy storage, and electric vehicles gives rise to dynamic, multi-objective optimization problems that are beyond the capabilities of rule-based controllers. Consumption patterns, price signals, ambient conditions, and user behavior can be exploited by data-driven methods based on deep learning and reinforcement learning to learn control policies with improved efficiency and cost-effectiveness [8]. This evolution reflects a move from reactive control towards predictive and decision-oriented operation of systems.

Even with these developments, deep learning is not easily translated into practical smart home systems. There are several reasons that make practical deployment of the system difficult, such as privacy and security issues associated with sensitive behavioral and health data [7], the high cost of collecting and annotating large amounts of data, and significant variation between households, which limits model generalization [5]. Also, there are limitations on the computational resources available on edge devices, which makes it difficult to implement real-time deployment of complex models [9]. Apart from this, some research has structural limitations. The existing literature is scattered and does not conform to standardized frameworks, and its applicability to various situations is not well validated. These factors hinder the development of smart home intelligence.

In response to these challenges, this paper is an application-oriented survey of smart homes and deep learning. It integrates the progress in the most significant application areas and methodological paradigms, as well as considers practical limitations and future research directions.

## 2. Major Application Domains of Deep Learning in Smart Homes

The use of deep learning has become common in smart home applications because the need to have contextual awareness, make intelligent decisions, and provide autonomous services is on the rise. In comparison with rule-based and shallow learning models, deep learning can represent heterogeneous, high-dimensional, and dynamically changing data more effectively, which facilitates tasks such as state understanding, activity recognition, risk detection, resource optimization, and natural human–machine interaction. Its purpose has therefore changed from improving individual perception modules to supporting the closed-loop paradigm of perception-understanding-decision-service.

The five most prominent areas of application that are already in existence can be categorized as follows: human activity recognition, health care and assisted living, smart energy control, security surveillance and anomaly detection, and voice recognition and intelligent control. These fields have their own task formulations, input sources, and methodologies, but they are inherently interconnected. Health monitoring and energy control are based on behavioral context through the use of activity recognition; security analysis is based on multimodal perception and contextual reasoning; and intelligent interaction is based on system understanding, which is then translated into user services. All these areas form the main functional components of deep learning-based smart home systems.

**Figure 1:**
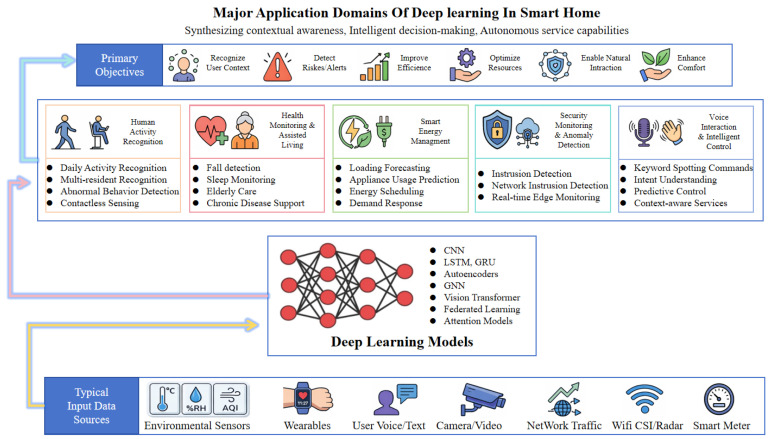
A unified framework of deep learning-driven smart home applications, highlighting the relationships among application domains, model paradigms, and multimodal data sources.

Figure 1 describes a general model of the interaction between application areas, models, and data. It is not an isolated module but a coupled system in which all the elements are interconnected. The framework emphasizes three points:

(1) the convergence of application fields and key aims, namely: context identification, risk identification, efficiency enhancement, and natural communication;
(2) the role of deep learning models (e.g., CNNs, RNNs, Transformers, and graph-based methods) in cross-domain intelligence;
(3) the combination of various sources of input data, such as environmental sensors, wearable devices, voice/text inputs, video streams, network traffic, WiFi/CSI, and smart meters.

This system-level analysis of the problem is important as it highlights that smart homes are not composed of isolated algorithms and systems, but rather of interrelated data, models, and application tasks. According to this model, Table 1 provides an overview of the major features of the five areas in terms of primary activities, input types, aims, and difficulties, which serves as a concise reference for understanding their roles and common limitations.

### 2.1. Human Activity Recognition

Human Activity Recognition (HAR) is one of the key tasks in smart homes, which links environmental perception and high-level decision-making. HAR offers valuable context to applications like health monitoring, anomaly detection, adaptive energy management, and personalized interaction by recording users’ activities, behavioral changes, and implicit intentions. It thus serves as a basic perception task and an important interface between low-level sensing and intelligent services.

**Figure 2:**
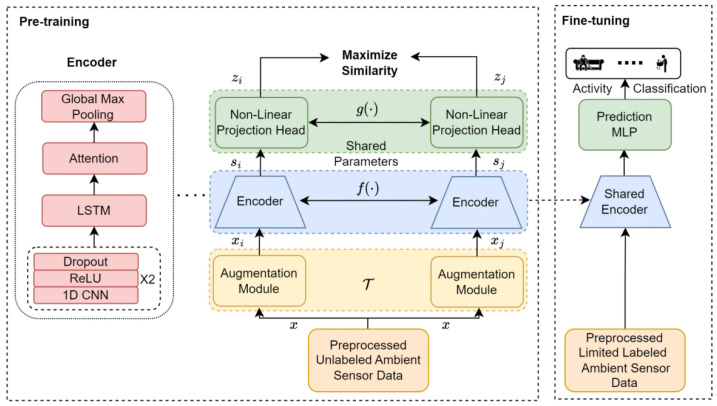
Architecture of a representative self-supervised human activity recognition framework (AttCLHAR), integrating contrastive learning, attention mechanisms, and temporal modeling. Adapted from Chen et al. [10].

In recent years, the trend of HAR in smart homes has shifted from well-controlled single activity classification to complex behavior modeling in real-life scenarios. The main reason behind this trend is that in-home behavioral data is often continuous, sparsely labeled, and expensive to annotate. Therefore, supervised learning methods have practical difficulties due to the lack of abundant labels. Self-supervised learning has been an active direction to improve data efficiency and generalization across environments. For instance, Chen et al. designed the AttCLHAR model, which combines the SimCLR contrastive learning framework with the attention mechanism and the CNN-LSTM hybrid architecture. As shown in Figure 2, the framework follows a two-stage pipeline, pre-training on unlabeled sensor data and then fine-tuning with few annotations, to obtain better performance under small-sample and weak supervision settings [10]. This trend shows a general shift in the HAR research community from seeking supervised accuracy to seeking robustness under realistic data conditions.

The second drawback is the lack of consideration of spatial context. Time-series data are not as efficient in capturing the temporal dynamics of the system, but they can be used to model the relationship between sensors and devices distributed across rooms. The solution to this problem is provided by graph-based approaches, where the relations among sensors are modeled explicitly. Srivatsa and Plötz proposed a HAR approach based on graphs with co-activation patterns and attention networks that capture the spatial dependencies in the environment [11]. This trend reflects a shift in activity recognition toward context-aware behavior understanding.

As the research progresses towards deployment, multi-resident scenarios and privacy issues become increasingly important. Ambiguity is introduced, and the robustness of the model decreases when there are overlapping activities across users. Moreover, centralized data collection poses privacy risks. In this context, Dahal et al. proposed the FeL-MAR framework, which integrates federated learning to allow for distributed training without sharing raw data, trading off between recognition performance and privacy protection [12]. This tendency indicates a general transition from offline accuracy optimization to deployment-related aspects such as data governance and multi-user adaptation.

From the sensing aspect, contactless perception has become a promising direction in recent HAR studies, which can improve usability, maintenance, and privacy. WiFi CSI-based approaches have been studied extensively due to their low deployment cost and device-free nature. Kang and Toh showed that a lightweight Attention-GRU model could obtain competitive recognition performance with highly efficient parameters and an edge-deployable model size [13]. Radar-based sensing, alternatively, is very attractive in privacy-concerned scenarios. Diraco et al. applied 60 GHz millimeter-wave radar with deep feature learning to recognize activities reliably in bathroom settings [14]. Tan et al. extended this work by combining micro-Doppler radar signals with 1D-CNN and BiGRU to learn fine-grained temporal-spectral patterns of human motions [15]. In general, we observe that the sensing modality for HAR is moving from conventional ambient sensors towards non-contact alternatives like WiFi and radar, to enable continuous, low-intrusive, and privacy-aware monitoring.

Recent work has also emphasized the implementation of HAR models under edge and IoT constraints. Biswas et al. [16] proposed a lightweight multimodal feature fusion and spatiotemporal learning framework for human action recognition on edge devices, demonstrating how multimodal representations can be optimized for resource-constrained inference. Similarly, Soman and Jeyaraj [17] developed an IoT-oriented HAR framework based on a hybrid ensemble classifier, integrating preprocessing, deep feature extraction, and temporal modeling components to improve activity recognition in smart environments. These studies indicate that HAR research is increasingly moving beyond offline recognition accuracy toward low-latency, resource-aware, and deployable models suitable for continuous smart home and health-monitoring applications.

HAR in smart homes is developing along three primary paths, namely from single-activity classification to continuous behavior comprehension, from fully supervised learning to data-efficient and context-sensitive modeling, and from contact-based sensing to contactless perception. Although these developments have been made, there are still major issues such as overlapping activities in multi-resident scenarios, cross-user variability, lack of generalization across environments, lack of rare-event data, and lack of interpretability. These problems still limit large-scale implementation in real-life scenarios.

### 2.2. Health Monitoring and Assisted Living

Health monitoring and assisted living represent one of the most impactful application domains of deep learning in smart homes, particularly in the context of population aging and the increasing demand for in-home care. Unlike human activity recognition, which focuses on instantaneous behavioral states, this domain emphasizes continuous modeling of long-term behavioral patterns, physiological dynamics, and health risks. The objective is to move from reactive event detection toward proactive and continuous risk management through pervasive sensing and data-driven analysis.

In the context of health monitoring, anomaly detection refers to the identification of deviations from an individual’s normal physiological, behavioral, or daily activity patterns. These deviations may indicate potential health deterioration, fall risk, irregular routines, or the need for assisted care, rather than malicious attacks or system-level security threats. In this context, it is useful to distinguish between point anomalies and collective or contextual anomalies. Point anomalies refer to sudden and isolated events, such as falls, abrupt inactivity, or emergency health-related incidents, which usually require immediate detection and response. In contrast, collective or contextual anomalies emerge from deviations accumulated over time or from mismatches between behavior and context. Examples include a gradual reduction in daily movement, irregular sleep–wake patterns, or repeated deviations from an individual’s normal routine, which may indicate functional decline or early health deterioration. Therefore, health-oriented anomaly detection in smart homes should not be limited to detecting sudden events, but should also model long-term behavioral baselines and temporal trends.

Anomaly detection constitutes a fundamental component of this paradigm, focusing on identifying deviations from habitual behavior over extended periods rather than isolated abnormal events. Such deviations often indicate early signs of health deterioration or safety risks. For example, Cejudo et al. developed a deep learning-based system that models population-level activity patterns to detect irregular behaviors in daily routines [18], while Rosca et al. integrated wearable and IoT data to capture changes in physiological and behavioral states [19]. These approaches highlight a shift from event-driven detection toward longitudinal and pattern-based health monitoring.

**Figure 3:**
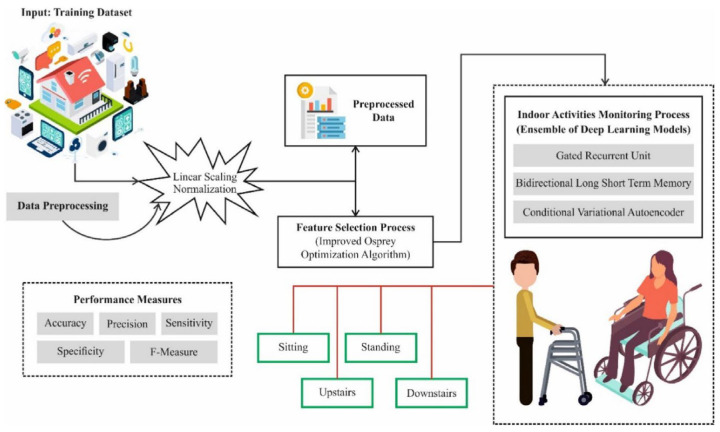
Deep learning-based indoor activity monitoring framework for assisted living and disability support. Adapted from Alsubaei et al. [21].

Building upon this foundation, deep learning has been increasingly applied to multimodal health monitoring systems that support long-term management and personalized care. Liu et al. combined improved Bayesian optimization with deep belief networks to develop a smart elderly care system that integrates multiple physiological signals, including body temperature, blood oxygen, blood pressure, heart rate, and physical activity, achieving favorable responsiveness and recognition performance [20]. Alsubaei et al. proposed an intelligent indoor monitoring framework for individuals with disabilities, employing BiLSTM, GRU, and conditional variational autoencoders to continuously model indoor activities and states [21]. As shown in Figure 3, such systems typically follow a pipeline involving data preprocessing, feature extraction, and multimodal inference, reflecting a transition from single-source sensing to integrated perception.

Voice and acoustic sensing are also emerging as promising data sources for health monitoring in smart homes. In addition to their conventional role in interaction and control, speech and non-speech acoustic signals can provide health-related cues, such as coughing, breathing patterns, sleep-related sounds, emotional changes, and potential indicators of cognitive or functional decline. When combined with wearable, environmental, and contactless sensing modalities, voice-based monitoring can enhance the continuity and coverage of assisted living systems. Nevertheless, these approaches also face challenges related to environmental noise, inter-individual variability, privacy protection, and the interpretation of health-relevant acoustic features.

Fall detection is one of the most important and well-known aspects of assisted living, as it applies to elderly individuals living alone or people with limited mobility. The current work on this subject has not only been aimed at enhancing the accuracy of fall detection but also at improving privacy protection and edge deployment capability. A method proposed by Cho et al. is a lightweight fall detection system that uses continuous-wave radar and binary neural networks, which is more accurate compared with other methods and can be implemented in resource-constrained systems due to the reduced model storage requirements [22]. Lupion et al. have further designed a privacy-aware system for fall detection and alerting based on wearable sensors and thermal imaging, which allows both detection and confirmation of events using voice assistance [23]. Simultaneously, there are increasing numbers of studies on millimeter-wave radar-based solutions, indicating the use of non-contact technologies in privacy-sensitive scenarios [24]. This trend is slowly developing into an area of research where it does not simply involve the identification of events, but the ability to identify, confirm, and respond to them.

Another important direction is automated food intake activity monitoring for nutrition-related health management. Food intake behaviors, including eating and drinking gestures, provide valuable information for dietary assessment, chronic disease management, and assisted living. Recent studies have shown that contactless sensing can support continuous and fine-grained monitoring of intake activities. For example, Wang et al. proposed Eat-Radar, an FMCW radar-based system that detects and segments eating and drinking gestures during meal sessions using a 3D temporal convolutional network with attention [25]. More recently, a robust multimodal learning framework combining contactless radar and wearable IMU sensors was developed to improve intake gesture detection and maintain robustness under missing-modality conditions [26]. These studies indicate that food intake activity monitoring can serve as an important bridge between human activity recognition and health-oriented smart home sensing.

Health monitoring in smart homes is progressively characterized by a transition towards continuous and context-aware human behavior modeling, where anomaly detection is embedded in daily routines instead of being event-based. On the other hand, sensing and analysis become naturally multimodal, combining physiological, behavioral, and contextual information to support comprehensive health assessment. As these systems approach real-world deployment, privacy preservation, computational efficiency, and long-term robustness are emerging as key issues in system development. Nevertheless, there are still some limitations that remain open. Firstly, the lack of abnormal health data limits the robustness of anomaly detection models. Secondly, significant inter-personal variability hinders generalization across subjects and environments. Finally, the absence of long-term real-world annotated datasets limits the validation and scalability of existing approaches.

### 2.3. Smart Energy Management

Smart energy management is one of the most practically relevant applications of deep learning in smart homes, directly supporting energy efficiency, cost reduction, and coordinated operation of increasingly complex residential energy systems. The increasing integration of distributed photovoltaics, energy storage, electric vehicles, and dynamic pricing mechanisms has introduced significant variability and multi-objective constraints that make traditional rule-based or static optimization approaches inadequate. In this context, recent research has shifted towards integrated frameworks combining load forecasting, system modeling, and adaptive control, with deep learning providing the basis for capturing temporal dynamics and decision-relevant system states [8,27].

**Figure 4:**
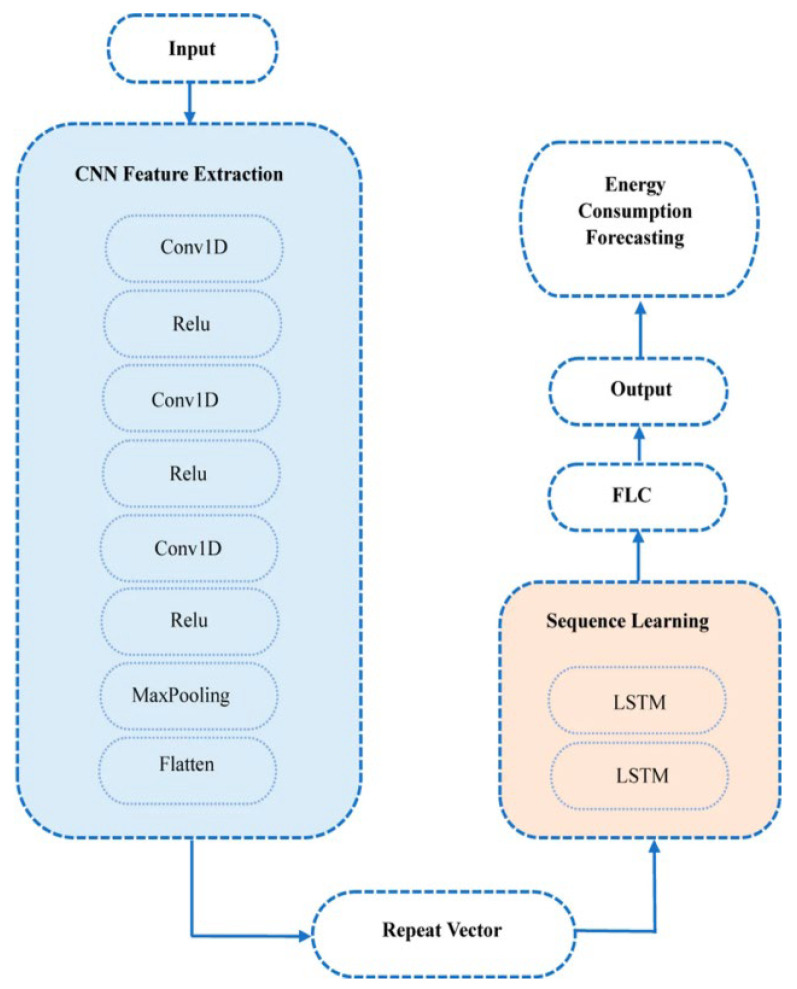
CNN–LSTM-based hybrid deep learning framework for short-term smart home energy consumption forecasting. Adapted from Ou Ali et al. [28].

Short-term load forecasting serves as a fundamental component of smart energy management. It is necessary to predict future energy consumption and power demand, enabling peak shaving, storage planning, and demand response strategies. The need to enhance the precision and reliability of load forecasting models has become a key research concern. In a comparison of ConvLSTM and CNN-LSTM architectures for household energy prediction, Ou Ali et al. showed that deep temporal networks are more effective, as they can capture time-series dependencies as well as local variations in energy consumption data [28]. As shown in Figure 4, this CNN-LSTM architecture first applies convolutional layers to learn local features before modeling temporal sequences with LSTM, which illustrates the integration of spatial feature extraction and sequence modeling. Cascone et al. have also developed a two-stage approach to electricity consumption prediction, where long-term forecasting using LSTM is combined with finer-grained predictions based on convolutional and recurrent neural networks [29]. Moreover, Rizwan et al. proposed a federated learning-based smart home energy prediction model, which allows distributed training without sharing raw household data, thereby addressing privacy concerns without compromising model accuracy [30].

The role of prediction is increasingly associated with decision-making as the reliability of forecasting improves. Forecasting outputs are no longer an isolated activity, but they are directly incorporated into control strategies in home energy management systems. In such environments, deep reinforcement learning (DRL) offers a natural framework to deal with dynamic environments and multi-objective optimization. Ben Slama and Mahmoud formulated energy scheduling as a Markov decision process and used reinforcement learning to maximize demand response when renewable integration was considered [8], whereas Xiong et al. added pricing signals and storage constraints to a unified DRL-based control system [27]. Multi-device coordination extensions such as appliances, storage systems, and vehicle-to-grid interactions further demonstrate a shift towards system-level control [31].

Smart home energy management is transforming into a system of integrated load forecasting that incorporates prediction, scheduling, and control. Instead of using fixed rules or offline optimization, recent trends favor data-driven methods, where reinforcement learning allows adaptive decision-making in dynamic and uncertain situations. Meanwhile, the scope of management is also extending beyond individual devices to system-level coordination, including user behavior, distributed generation, energy storage, and grid interaction. However, it is nontrivial to translate these advances into real-world deployment. The performance of models can be degraded across different households because of poor generalization, and user comfort is difficult to quantify and incorporate into optimization objectives. In addition, real-time implementation on edge devices is limited by computational resources and system cost, which pose practical constraints on large-scale adoption.

### 2.4. Security Monitoring and Anomaly Detection

The security of smart home systems is also important in ensuring that these systems are reliable and safe. With the integration of heterogeneous devices such as cameras, smart locks, voice assistants, IoT sensors, etc., the threat surface has expanded, evolving from intrusion detection to include abnormal user activities, device failures, cyber threats, and data loss. Security solutions are therefore shifting towards more proactive and reactive alerts, as well as more active risk monitoring and mitigation, using deep learning to process signals in highly heterogeneous environments.

In contrast to health-oriented anomaly detection, anomaly detection in security monitoring focuses on abnormal physical events, device behaviors, and network traffic patterns that may indicate intrusions, unauthorized access, cyberattacks, device malfunction, or other safety threats. Most security-related anomalies can be regarded as point anomalies when they involve sudden events, such as unauthorized entry, abnormal device access, or abrupt changes in network traffic. However, collective or contextual anomalies may also occur in security scenarios when multiple weak signals jointly indicate a risk, such as repeated unusual access attempts, persistent deviations in device telemetry, or abnormal usage patterns that are only meaningful relative to time, location, or user context. This distinction suggests that security monitoring should combine real-time event detection with temporal and contextual reasoning. Therefore, although the same term is used, its objective, data sources, and risk implications differ from those in health monitoring.

Vision-based abnormality detection of the physical environment is still one of the most traditional methods in this area, focusing on intrusions and suspicious behaviors. The combination of deep learning systems with cameras and sensors has been found to be effective at identifying abnormal motion patterns in real time [32]. Nonetheless, this is not a purely visual approach, as it faces limitations such as occlusion, illumination changes, and privacy concerns, which restrict its applicability in real-world scenarios. These limitations have led to increasing interest in multimodal sensing approaches that are not strictly limited to visual inputs.

Meanwhile, network and device security issues have become more prominent. IoT devices are particularly vulnerable due to anomalies in communication, unauthorized access, and coordinated attacks on the system. In this regard, anomaly detection has shifted towards data-driven approaches that model network behavior, rather than relying on rule-based or signature-based methods. Du et al. introduced an innovative intrusion detection model based on a combination of convolutional feature extraction and global attention mechanisms (MBConv-ViT) [33], and Bajpai et al. further improved performance through enhanced neural network training strategies [34]. These solutions reflect a broader shift toward representation learning-based security analysis.

**Figure 5:**
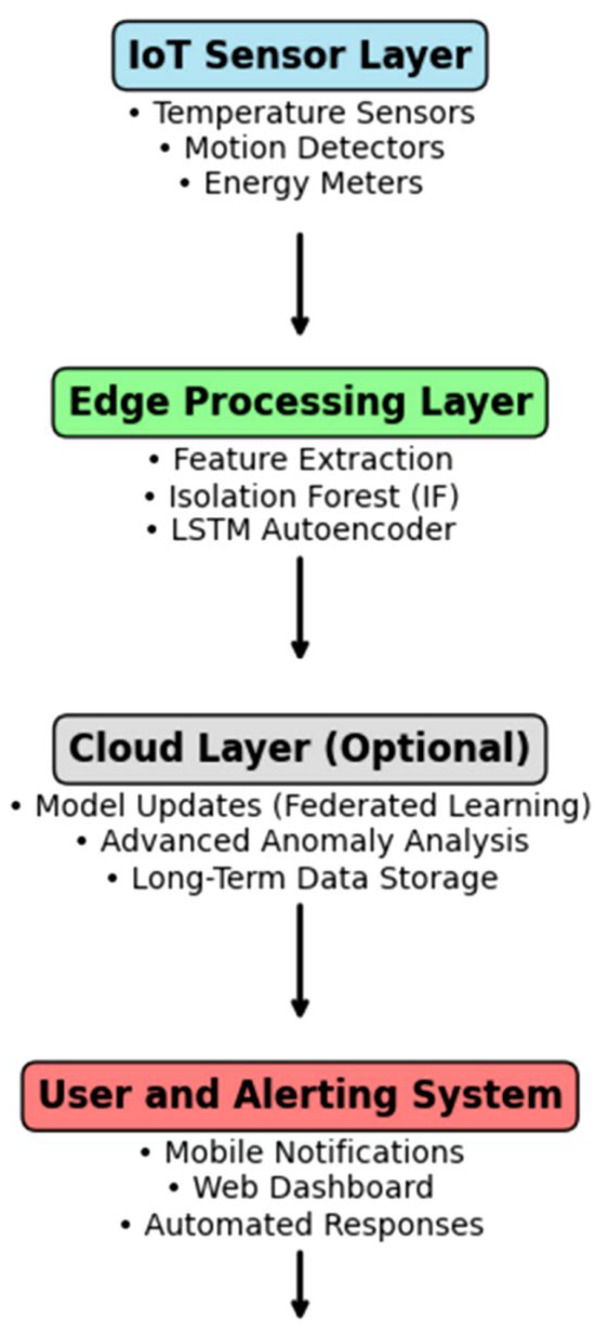
Edge–AI framework for real-time anomaly detection and alerting in smart home environments. Adapted from Reis et al. [35].

Deployment considerations are another factor in the evolution of security architectures. Edge intelligence enables real-time processing, low latency, and continuous operation, supporting effective monitoring. Reis et al. proposed an Edge–AI framework that combines multimodal sensing with lightweight anomaly detection models for resource-constrained systems [35]. The system shown in Figure 5 integrates sensing, edge processing, and alerting within a single pipeline, reducing reliance on cloud infrastructure and enhancing both responsiveness and privacy.

In addition, recent work has been devoted to the solution of cross-modal and platform-agnostic approaches to the problem of device heterogeneity in a real-world situation. These solutions allow non-intrusive monitoring of smart and non-smart devices by integrating complementary data sources like audio signals and network traffic [36]. Simultaneously, there is a tendency towards security analysis which goes beyond the concept of detecting anomalies but instead multi-level reasoning where the detection, localization and root cause analysis are put into one framework [37]. This change is an indication that the focus on system-level observability and interpretability is growing.

With the combination of these trends, it can be said that smart home security is being monitored in a more physical manner and in the network layer. It is no longer an isolated event but rather a detection process that is integrated into the continuous monitoring processes and is supported by multimodal inputs and dynamic models. Meanwhile, the pressure to put models at the edge and make them privacy-conscious is the factor that creates new limitations on model complexity and data consumption. The current problems still exist, such as high false alarm rate in multi-complex environments, lack of generalization between different types of homes, and the problem of trade-off between the efficiency of security measures and the protection of personal information.

### 2.5. Voice Interaction and Intelligent Control

The voice and smart control will be the key to making the smart homes not only a system of automation but also an environment that is user-friendly and facilitates natural interaction with the user. The modalities of voice, gestures, and gaze are much more interactive than traditional interfaces like mobile applications or fixed controls and can be used in more flexible ways when performing tasks on a daily basis. In its turn, as deep learning has developed, the process of interaction between users and smart homes has ceased to be based on the performance of direct commands and has become aimed at the interpretation of user intentions and providing services in accordance with the situation.

**Figure 6:**
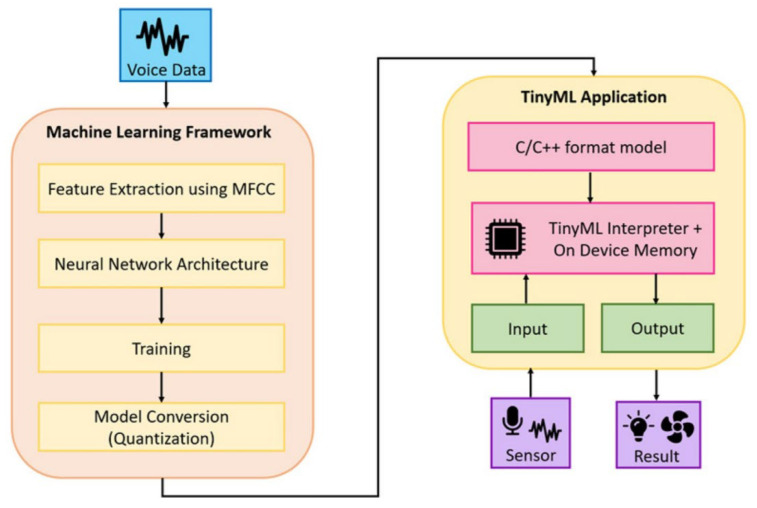
TinyML-based keyword spotting and on-device voice control framework for smart homes. Adapted from Malche et al. [38].

At the level of basic interaction, recent works focus on efficient and on-device processing with constrained resources. Lightweight keyword spotting and speech recognition models based on TinyML have made real-time voice control possible on embedded devices, alleviating dependency on cloud infrastructure and enhancing response speed and privacy [38,39]. Such systems often follow a pipeline from feature extraction to model deployment, as shown in Figure 6, demonstrating the possibility of edge-based voice interaction.

Interaction is, however, not merely a matter of command recognition in real-life situations, as user input tends to be ambiguous and context-dependent. This has brought about growing interest in intent understanding, which aims to resolve ambiguity and guide system responses through multimodal information. A framework that Calo et al. suggested was the use of language with contextual information in order to disambiguate multimodal data in an interactive manner to help interpret commands given by users [40]. These approaches show that there is a change from direct command mapping toward more contextual and bidirectional interaction.

Beyond command recognition and intent disambiguation, recent studies have increasingly emphasized affect-aware interaction in companion-style smart home systems. Multimodal emotion recognition enables smart environments to infer user affective states from complementary cues such as facial expressions, speech signals, and contextual behavior. For example, Lu et al. [41] proposed a lightweight multimodal emotion recognition framework for companion robots, integrating facial and speech features within a deep learning architecture to support real-time affective perception. Such systems suggest that smart home interaction is evolving from recognizing explicit commands toward understanding users’ emotional and social states, thereby enabling more empathetic, proactive, and human-centered services.

This development is further extended to multimodal interaction frameworks that integrate voice with supplementary signals like gaze, gestures, and spatial context. Mixed-reality interfaces and radar-based gesture recognition systems illustrate the possibility of combining multiple modalities in order to infer user intent and enhance interaction flexibility [42,43]. These developments indicate that voice is becoming more integrated into a wider interaction ecosystem instead of serving as an independent control channel.

On the system level, there is a change in control modes toward behavior-based and proactive mechanisms rather than reactive ones. Intelligent systems can be used to model user habits as well as situational patterns and use that information to anticipate user needs and adjust device states accordingly [44]. This will lead to a new paradigm shift from interaction-based automation to predictive and adaptive automation, in which control methods are not only guided by explicit inputs but also by implicit behavioral signals.

With the development of these capabilities, smart home interaction is becoming more closely related to perception, reasoning, and control processes. Instead of functioning as a standalone interface, voice interaction is now being incorporated into system-level pipelines that integrate multimodal sensing, intent inference, and adaptive control. At the same time, several challenges remain. In open and noisy environments, speech recognition is still difficult, especially when it comes to unconstrained language input. Semantic alignment is complicated by interoperability across heterogeneous devices, and greater automation levels raise concerns about user control, transparency, and trust. These factors continue to affect the usability and scalability of intelligent interaction systems in real-world applications.

## 3. Deep Learning Methods and Technical Evolution in Smart Homes

Deep learning has become a core methodological foundation in smart home research, supporting a wide range of tasks, including perception, recognition, reasoning, prediction, and decision-making. As smart home scenarios have become increasingly complex, the role of deep learning has also evolved. Early studies mainly relied on convolutional and recurrent architectures to extract local features and model temporal dynamics from sensor data. More recent studies have introduced Transformer-based models, graph learning, multimodal fusion strategies, and deep reinforcement learning to address long-range dependencies, relational reasoning, heterogeneous data integration, and adaptive control.

This methodological evolution is closely associated with the transformation of smart home systems. Model design has shifted from recognizing isolated patterns to modeling interactions among users, devices, sensors, and environments. Meanwhile, input data have evolved from single-modality streams to heterogeneous multimodal data that integrate environmental sensing, behavioral signals, physiological information, acoustic cues, visual data, and device-level states. Accordingly, research objectives are no longer limited to classification and prediction, but increasingly extend to scheduling, control, optimization, and human-centered services. These changes indicate a transition from isolated perception models toward integrated smart home intelligence pipelines that combine perception, understanding, decision-making, and deployment.

To avoid treating heterogeneous methods as mutually exclusive model categories, this section organizes representative deep learning paradigms according to their functional roles in smart home intelligence pipelines. CNN-, RNN/LSTM/GRU-, Transformer-, and graph-based methods mainly serve as representation and reasoning architectures, focusing, respectively, on local feature extraction, temporal modeling, global context learning, and relational reasoning. Multimodal fusion is treated as a cross-modal integration strategy that combines heterogeneous sensing sources, while deep reinforcement learning is regarded as a decision-making and control paradigm for adaptive system operation. Therefore, these paradigms should be understood as complementary functional components rather than strictly independent architectural classes.

Based on this view, Table 2 summarizes representative deep learning paradigms in terms of their functional roles, core strengths, typical applications, representative inputs, limitations, and deployment considerations. Rather than providing a rigid taxonomy of mutually exclusive models, the table highlights how different paradigms contribute to different layers of smart home intelligence, including perception, representation, reasoning, fusion, and control.

The comparison in Table 2 shows that these paradigms are not mutually exclusive. In practical smart home systems, they are often combined according to task requirements and deployment constraints. For example, CNNs may be used as local feature encoders, recurrent or Transformer-based models may capture temporal or global dependencies, graph models may represent sensor-device relationships, multimodal fusion may integrate complementary information, and deep reinforcement learning may support adaptive control. This reflects a broader shift from model-centric development toward system-oriented design, where methodological choices are guided by data characteristics, functional roles, and real-world deployment requirements.

### 3.1. CNN-Based Methods

Convolutional Neural Networks (CNNs) are among the first and most popular families of models in smart home research, which are mainly used as local representation learning tools. They have local receptive fields and share parameters that allow them to efficiently extract patterns that are localized in space and are robust to noise and variations in input. This renders CNNs especially appropriate for heterogeneous smart home data such as sensor streams, energy consumption sequences, WiFi and radar signals, and visual inputs, where local structure is frequently informative.

Another aspect of activity recognition is the ability to convert non-spatial sensor data into a form that can be used in convolutional processing. CNN-based models can be trained on raw sensor events and then converted into structured or image-like forms, which allow them to better understand spatial distribution and local activity patterns. As an example, Song and Wang have translated sensor information into RGB activity images and implemented convolutional attention mechanisms to enhance recognition performance [45], whereas Agac et al. have implemented attention modules with lightweight convolutional-temporal systems to reduce the accuracy-efficiency trade-off under resource constraints [46]. Such methods show how significant it is to create appropriate representations when using CNNs to process non-visual information.

CNNs are also at the center of modeling energy consumption, especially in terms of short-term variations and local time-series patterns. Convolutional layers are not used independently but instead combined with recurrent structures to create hybrid models that make use of both local and sequential dependencies. Ou Ali et al. have shown that ConvLSTM and CNN-LSTM models can be successfully applied to combine these capabilities for short-term energy forecasting [28], and hybrid CNN-LSTM models have also been applied in energy consumption prediction to extract spatial features with convolutional layers and capture temporal dependencies with LSTM layers, demonstrating improved prediction performance on multivariate datasets [47]. CNNs in such environments serve as part of spatiotemporal modeling pipelines rather than independent predictors.

CNN-based techniques are also being used in interaction and contactless sensing applications with emerging sensing modalities such as radar and WiFi signals. Chen et al. have applied convolutional feature extraction to recognize gestures using millimeter-wave radar point clouds [43], whereas Tan et al. have combined 1D-CNN and recurrent models to capture micro-Doppler signal properties [15]. These examples illustrate the versatility of CNNs across various forms of signal representation, as well as their role in enabling non-contact interaction and perception.

Methodologically, the function of CNNs has been slowly changing into modular units of more complicated architectures. Their performance is no longer based solely on the extraction of local features but also on how they are combined with temporal modeling, attention mechanisms, and multimodal processing. With the growing need to model long-range dependencies and contextual relationships in smart home applications, CNNs are seldom used as standalone systems. Rather, they act as efficient front-end encoders that provide structured representations to higher-level reasoning and decision-making modules.

### 3.2. RNN/LSTM/GRU-Based Methods

Recurrent Neural Networks (RNNs) and other variants, such as Long Short-Term Memory (LSTM) and Gated Recurrent Unit (GRU), are the main methods used to model temporal dynamics in smart home systems. Unlike convolutional models, which rely on the ability to find local patterns, recurrent models are designed with explicit sequential dependencies, making them suitable for data characterized by continuity over time, such as user behavior, environmental changes, and energy consumption. In this way, they serve as one of the most important links between perception-level representations and higher-level state interpretation.

RNN-based models are used in activity recognition to overcome the limitations of purely local feature extraction by modeling the temporal evolution of activity sequences. The combination of convolutional encoding with recurrent layers has become a popular design pattern for hybrid architectures to fuse spatial and temporal representations. For instance, Chen et al. included LSTM in a self-supervised framework to alleviate the limited annotation problem [10]. A lightweight Attention-GRU model demonstrates a good balance between efficiency and accuracy in WiFi-based sensing [13]. Related architectures have also been used in assisted living systems for continuous monitoring of behavioral and physiological states, which demand robust temporal modeling [21].

The extension of RNN-based models to anomaly detection is straightforward when deviations are related to temporal patterns rather than static features. Learning normal behavior dynamics over long periods allows LSTM and GRU-based architectures to detect even slight irregularities in physical and network-level data. Edge-based anomaly detection systems [35] and intrusion detection models for IoT environments [48] utilize this ability of RNNs to capture temporal baselines in dynamic systems.

RNN-based methods are used in energy management to model periodicity, trends, and short-term variability of consumption data. They are especially applicable to forecasting and control tasks due to their capability to capture multi-scale temporal dependencies. GRU- and BiLSTM-based frameworks have been used to forecast energy usage, cost, and emissions in residential settings [49], and similar models support predictive control of heating and cooling systems [50]. Recurrent architectures serve as core components in these applications to represent system dynamics over time.

In terms of methodology, the role of RNN-based models is also evolving with the growing complexity of systems. Architectural evolution has been driven by the integration of bidirectional structures, attention mechanisms, and autoencoder designs, which enhance their ability to model intricate temporal relationships. Meanwhile, they are increasingly combined with other model families, as single-sequence models are gradually being replaced by modular components within larger pipelines.

Although the popularity of Transformer architectures is increasing, RNN-based models still play a role when computational efficiency and stable sequential modeling are required. The limitations of RNNs, such as the inability to capture long-range dependencies and limited support for parallel computation, have led to the development of hybrid approaches that integrate recurrent structures with convolutional, attention-based, or graph-based modules. In this way, RNNs are applied as intermediate temporal encoders in integrated systems rather than as end-to-end solutions.

### 3.3. Transformer-Based Methods

As smart home systems increasingly require long-term behavior understanding, multimodal data integration, and context-aware reasoning, Transformer-based models have emerged as a natural extension of sequence modeling approaches. Unlike recurrent architectures that process data sequentially, Transformers rely on self-attention to capture dependencies across all time steps and modalities simultaneously, enabling more flexible modeling of global context. This capability is particularly relevant in smart home environments, where user behavior, environmental signals, and device states are tightly coupled over extended temporal horizons.

In activity recognition, Transformer-based approaches improve the modeling of multi-sensor sequences by capturing long-range dependencies and cross-sensor interactions. Huang et al. demonstrated that unified encoding of multi-sensor data can enhance the recognition of complex activities [51], while Transformer models applied to WiFi CSI signals further illustrate their ability to represent spatiotemporal patterns beyond local or sequential constraints [52]. These developments indicate that Transformers are not merely alternatives to recurrent models, but also provide a more expressive framework for global representation learning.

Their applicability extends beyond perception tasks to energy modeling and interaction. In energy forecasting, Transformer-based models effectively handle multi-scale temporal variability and incorporate exogenous factors such as weather and appliance usage [53]. In interaction scenarios, language-based Transformers enable more robust semantic understanding of user commands, supporting flexible and context-aware interpretation [54,55]. This expansion reflects a shift from sensor-level modeling toward unified representations that combine temporal, contextual, and semantic information.

However, the advantages of Transformers come with increased computational demands, posing challenges for deployment in resource-constrained environments. Recent efforts therefore focus on improving efficiency through model compression, pruning, and lightweight design. Studies on embedded platforms indicate that while standard architectures remain resource-intensive, optimized variants can achieve acceptable performance for edge deployment [56]. This highlights the importance of balancing representational capacity with practical constraints.

From a broader perspective, Transformer-based methods are reshaping methodological design in smart homes by enabling unified modeling across time, modalities, and context. At the same time, their effectiveness depends on data scale and task complexity, and they may not always provide advantages in simpler or resource-limited scenarios. In practice, they are increasingly combined with convolutional or recurrent components, forming hybrid architectures that integrate local, temporal, and global modeling capabilities within a single system.

### 3.4. Graph Learning and Multimodal Fusion Methods

Modeling relationships between users, devices, and environmental factors has become as important as modeling temporal dynamics as smart home environments become more complex. The two complementary directions that tackle this issue in different ways include graph learning and multimodal fusion. The former is based on the explicit representation of structural dependencies, whereas the latter focuses on combining heterogeneous sensory information to enhance robustness and coverage.

Graph-based techniques can be used to represent interactions between sensors, devices, and users. They capture the spatial structure of co-activation among entities as well as spatial configurations, enabling behavior modeling beyond pure temporal analysis. As an illustration, graph neural networks have been applied to predict sensor interactions and enhance activity recognition in multi-room settings [11], as well as attention-based graph models, which further refine the representation of spatiotemporal relationships [57]. In addition to sensing, human-object interaction analysis and device coordination are also modeled using graph structures, which help represent daily activities and system behavior in more realistic terms [58].

Simultaneously, multimodal data fusion has been developed to overcome the limitations of single-channel sensing, allowing information from multiple sources (vision, audio, radar, and network data) to be integrated. Multimodal integration in interaction tasks enhances intent understanding when input is ambiguous or incomplete [40]. In perception tasks, the combination of radar, WiFi, and acoustic signals has been shown to improve robustness against occlusion, noise, and environmental variation [59,60]. Attention-based fusion approaches are better suited to heterogeneous and dynamic smart home systems than fixed fusion strategies, as they enable adaptive weighting across modalities.

Resident-specific feature extraction is another important direction enabled by multimodal fusion, particularly in multi-resident smart home environments. Conventional activity recognition models often focus on activity labels alone, but they may fail to distinguish whether similar activities are performed by different residents. By combining complementary sensing sources, multimodal fusion can support resident-aware representation learning. For example, WiFi CSI or radar signals can capture device-free motion and spatial activity patterns, while wearable sensors can provide user-specific inertial, physiological, or identity-related cues. Such integration allows smart home systems to learn representations that are both activity-aware and resident-aware, thereby supporting multi-resident activity recognition, personalized health monitoring, and adaptive smart home services. This direction is also closely related to the generalization and transferability challenges discussed in Section 5.3, since cross-user variability remains a major source of performance degradation in real-world deployment.

In addition to improving activity recognition and intent understanding, multimodal fusion also plays an increasingly important role in affective and socially aware smart home interaction. By combining facial, acoustic, behavioral, and contextual signals, multimodal models can infer not only what users are doing or requesting, but also how they feel and whether assistance may be needed. Lightweight multimodal emotion recognition frameworks for companion robots demonstrate that facial and speech features can be integrated for affective perception under practical deployment constraints. Similarly, lightweight spatio-temporal attention networks for video anomaly detection show how efficient visual representation learning can support the identification of abnormal or socially relevant events [61]. These studies indicate that multimodal fusion is shifting from low-level signal integration toward higher-level interpretation of user intention, emotional state, and contextual risk.

These directions tend to converge in high-level applications. Graph-based representations can be combined with multimodal inputs for relational reasoning, recommendation, and system-level decision-making, such as in knowledge graph-based energy management frameworks [62]. Such approaches represent a shift from state recognition to relational understanding and informed decision support.

This methodological shift reflects a broader trend from implicit dependency modeling to explicit representation of structure and context. Input data are becoming increasingly heterogeneous, and task objectives extend beyond classification to include reasoning, coordination, and control. Consequently, graph learning and multimodal fusion should be viewed as complementary components within complex systems rather than as standalone methods.

These strategies are not without limitations. Graph-based methods require high-quality graph construction and updating, while multimodal fusion must address challenges such as synchronization, missing modalities, and computational overhead. In cases where data are sparse or modality quality is poor, performance gains may become unstable. These challenges highlight the importance of careful system design in real-world smart home applications.

### 3.5. Deep Reinforcement Learning for Decision-Making and Control

Deep reinforcement learning (DRL) is an approach in smart home systems that differs in its focus on decision-making rather than representation or prediction. CNN, RNN, and Transformer-based models are mostly used to assist perception and state estimation, whereas DRL focuses on learning control policies through interaction with dynamic systems. It is especially important in the context of smart homes because system behavior is influenced by the combination of user actions, device states, environmental conditions, as well as external signals such as energy prices. In such contexts, DRL allows formulating control as a decision process with long-term objectives and feedback signals.

Home energy management is the most well-known application domain of DRL because it inherently involves dynamic scheduling in the presence of uncertainty and competing objectives. DRL-based methods can learn policies that trade off between energy cost, user comfort, and operational constraints by formulating scheduling tasks as Markov decision processes. For example, reinforcement learning has been applied to demand response optimization and storage control, showing better adaptability than rule-based or offline optimization [8,27]. In these situations, DRL not only produces effective optimization results but also adapts policies continuously in response to changing conditions.

In the recent literature, this concept has been further extended by incorporating human behavior into the decision process. Optimization of device states is no longer performed in isolation, but DRL-based systems increasingly utilize outputs from perception modules (such as activity recognition) to guide control policies. This enables context-sensitive decision-making, where system actions are adjusted based on user behavior and environmental conditions [63]. This combination reflects a shift from device-oriented optimization to user-oriented and situation-dependent regulation.

At a larger scale, DRL is also being studied in the context of personalization and inter-household variation. Federated or distributed reinforcement learning enables policy learning across multiple households while preserving local adaptation [64]. This indicates growing interest in balancing generalization and personalization, as control mechanisms must account for heterogeneous household structures and user preferences. In such frameworks, DRL serves not only as an optimization method but also as a core decision-making framework for adaptive smart home systems.

The methodological principles of DRL can be applied to a broader set of control tasks beyond energy management, such as indoor climate regulation, device coordination, and environment-aware automation [65]. These applications share similar characteristics, where system states evolve over time and decisions must account for delayed and cumulative effects. In this way, DRL provides a unified framework for modeling such control processes across diverse scenarios.

From a system perspective, DRL represents a shift toward decision-centric architectures rather than perception-centric pipelines. Rather than treating prediction as the final output, policy learning becomes an integral component of smart home systems, enabling closed-loop interaction between sensing, inference, and control. This shift redefines the role of deep learning in such systems, extending it from state estimation to active control of future system behavior.

Despite its potential, DRL also has notable limitations. Learning effective policies typically requires long training periods and is difficult to implement in real-world settings due to safety and usability concerns. Challenges such as sample efficiency, training stability, reward design, and interpretability further complicate deployment. In addition, computational requirements and system complexity may limit feasibility in resource-constrained environments. These factors make DRL more effective when integrated with predictive models, simulation environments, and edge-aware architectures rather than being used as a standalone solution.

### 3.6. Federated Learning for Privacy-Preserving Distributed Intelligence

Federated learning has become an increasingly important learning paradigm in smart home research because household data are naturally distributed, privacy-sensitive, and highly heterogeneous. Unlike centralized learning, which requires raw data from different homes or devices to be uploaded to a cloud server, federated learning enables multiple local clients to collaboratively train a shared model while keeping raw data on local devices. This property makes it particularly suitable for smart home scenarios involving behavioral routines, physiological signals, energy consumption records, and network traffic, where direct data sharing may raise serious privacy and security concerns.

In smart home applications, federated learning has been explored in several representative domains. For human activity recognition, federated learning can support multi-resident and cross-household model training without exposing raw activity data [12]. In smart energy management, federated and transfer learning approaches allow households to share useful consumption patterns while preserving local energy records [30]. For security monitoring and intrusion detection, federated learning can enable distributed anomaly detection across heterogeneous IoT devices and home networks while reducing the risks associated with centralized data collection. Federated learning is not only a privacy-preserving technique but also a practical framework for improving model generalization across distributed smart home environments.

Nevertheless, federated learning also introduces new challenges. Smart home data are often non-independent and identically distributed because different households have different layouts, devices, routines, and user preferences. This non-IID property can reduce convergence stability and model performance. In addition, communication cost, client availability, device heterogeneity, model poisoning, and privacy leakage from model updates remain important issues. Therefore, future federated smart home systems should integrate privacy-preserving training, robust aggregation, personalization, and edge-aware deployment to balance privacy protection, model accuracy, and system efficiency.

## 4. Public Datasets and Evaluation Metrics in Smart Homes

### 4.1. Overview of Public Datasets

Beyond model design, the development of deep learning in smart homes is also highly dependent on the availability of public datasets and the coherence of evaluation protocols. In comparison to single-modality domains, research in smart homes spans a wider variety of data sources such as ambient sensing, wearable signals, visual monitoring, energy consumption, network traffic, and speech interaction. This leads to higher complexity in terms of data representation and benchmarking. Thus, a survey of representative datasets (Table 3) is crucial to understand the empirical basis of current work and the comparability of reported results.

Human activity recognition remains the most mature and data-rich area in this field. Early datasets were primarily based on ambient sensors and inertial measurements, with CASAS and UCI HAR serving as widely adopted benchmarks [1,4]. As research has shifted toward realistic and complex in-home scenarios, dataset design has expanded to include multimodal sensing, vision-based monitoring, and privacy-preserving modalities such as thermal imaging. Datasets such as Toyota Smarthome, UP-Fall, and eHomeSeniors reflect this transition, capturing increasingly diverse and context-rich activity patterns [66,67,68]. This progression indicates a gradual movement from controlled benchmarks toward more realistic and application-oriented data environments.

In contrast, datasets for health monitoring and assisted living remain relatively limited. The collection of such data often involves sensitive information, including falls, abnormal behaviors, and long-term physiological changes, which increases both acquisition cost and annotation difficulty. Although existing datasets support specific tasks such as fall detection and privacy-aware monitoring [67,68], large-scale, long-duration, and multi-resident datasets with sufficient diversity are still scarce. This limitation restricts the ability to evaluate model robustness and generalization in real-world care scenarios.

Energy-related datasets exhibit a different structure, focusing on continuous measurement and temporal dynamics. Resources such as REDD, UK-DALE, and REFIT provide detailed records of household electricity consumption at both aggregate and appliance levels [69,70,71]. These datasets are particularly suited for studying load forecasting, demand response, and occupancy inference, where long-term temporal patterns and user–device interactions play a central role. Compared with activity datasets, they emphasize continuity and system-level dynamics rather than discrete events.

For security monitoring and anomaly detection, widely used datasets are often derived from broader IoT or network security contexts rather than fully realistic residential settings. Datasets such as TON_IoT and CICIoT2023 provide valuable benchmarks for intrusion detection and anomalous traffic analysis [72,73]. However, their scenario composition and device distributions do not always reflect real household environments, which limits the direct transferability of experimental results to practical deployments.

In the domain of voice interaction, public datasets primarily focus on speech recognition and intent understanding. Benchmarks such as Speech Commands and Fluent Speech Commands support keyword spotting and semantic parsing tasks [74,75]. While these datasets are important for developing interaction models, they typically isolate speech signals from the broader context of home environments, lacking multimodal cues and real-world noise conditions.

Overall, existing datasets cover the major application areas of smart homes but exhibit significant differences in scale, realism, modality coverage, and temporal span. These inconsistencies make it difficult to compare results across studies and may limit the interpretability of reported performance. In particular, the lack of high-quality datasets for health monitoring, multi-resident scenarios, and realistic anomaly detection remains a critical barrier to advancing the field.

The comparison also shows that model development does not always keep pace with the development of data quality and benchmarking practices. With models growing more complicated and data-hungry, the lack of standardized, large-scale, and realistic datasets increasingly limits reliable evaluation. Addressing this imbalance will be necessary to enable a shift from method-driven improvements to system-level validation in real-world smart home settings.

### 4.2. Evaluation Metrics

The diversity of task objectives and system requirements in smart home research has an intrinsic impact on evaluation. In the works discussed in the current reference set, there is no single universal criterion around which evaluation is organized, but rather a set of task-dependent metric groups. Studies on activity and behavior recognition are primarily evaluated using classification measures, forecasting studies use regression error metrics, control studies based on reinforcement learning rely on operational outcome indicators, and edge deployment studies adopt efficiency-oriented measures. This reflects the reality that smart home systems tend to integrate perception, prediction, decision-making, and deployment under resource constraints, rather than solving a single standalone problem.

For classification tasks (e.g., human activity recognition, gesture recognition, and similar behavior understanding), the most frequently reported metrics are Accuracy, Precision, Recall, and F1-score [10,11,13,15], as seen in studies on ambient-sensor HAR, graph-based HAR, WiFi CSI-based recognition, and radar-based recognition. The F1-score is especially useful when both false positives and false negatives must be considered, such as in multi-class or imbalanced activity scenarios, compared to using accuracy alone.

In health event detection, particularly fall detection, validated studies in the reference set tend to report Accuracy, Sensitivity (or Recall), Specificity, and Precision [22,24]. This focus aligns with the safety-critical nature of the task, as both missed detections and false alarms directly affect system reliability and user acceptance in assisted living settings.

Accuracy, Precision, Recall, and F1-score [33,34,35] are also commonly used in anomaly detection and intrusion/security monitoring. The central issue in this area is not only whether abnormal events can be identified, but also whether they can be reliably distinguished from normal smart home activity without excessive misclassification. This metric combination is more strongly supported by the studies in the current reference set than the more generic descriptions often found in broader surveys.

In regression tasks, such as forecasting household energy consumption and short-term power demand, performance is typically evaluated using MAE, RMSE, and MAPE [28]. These metrics provide complementary perspectives: MAE reflects average absolute deviation, RMSE is more sensitive to large errors, and MAPE facilitates comparison across different consumption levels. This error-based evaluation framework is more directly supported by the verified forecasting studies you have included than classification-style metrics.

For decision-making tasks, especially those based on deep reinforcement learning for smart home energy management, the focus shifts from predictive accuracy to operational effectiveness. Confirmed studies emphasize outcomes such as electricity cost minimization, user or thermal comfort, and in some cases revenue from participation in ancillary services [27,63,64,65]. This category is therefore better characterized by control-oriented performance indicators rather than traditional prediction metrics alone.

Beyond task-specific performance, deployment-oriented evaluation is becoming increasingly important in smart home research. In edge and TinyML-related studies within the current reference set, commonly reported indicators include inference latency, RAM/memory footprint, flash/storage footprint, number of parameters, and FLOPs [38,39,46]. A model that performs well offline may still be impractical if it cannot operate efficiently on low-power embedded or edge devices.

The most directly supported evaluation metrics in the current reference set are summarized in Table 4. This task–metric mapping more closely reflects how cited smart home studies evaluate model performance in practice, rather than providing a generic inventory of metrics.

The comparison results show that there is no single performance measure in smart home research, but rather a trade-off between system accuracy and robustness, computational efficiency, and practical applicability. Evaluation measures are not only a means of assessing performance, but also constraints on model design, system optimization, and deployment.

When combined with the dataset properties discussed in Section 4.1, current evaluation practices reveal a gap between methodological development and empirical validation. While model architectures become increasingly sophisticated, the poor quality of datasets, unrealistic scenarios, and inconsistent metrics still undermine the robustness, comparability, and reproducibility of reported results. Closing this gap requires more realistic benchmarks, task-aware evaluation methodologies, and reporting guidelines that bridge experimental performance with real-world applicability in smart home deployments.

## 5. Challenges and Future Directions

### 5.1. Data Challenges

The constraints on data are still a bottleneck in smart home deep learning research. Unlike computer vision or natural language processing, where large-scale datasets can be easily collected and standardized, smart home data are inherently continuous, multi-source, and context-dependent, which requires long-term deployment in real residential environments. The high cost of deployment, privacy issues, and the need for continuous monitoring make it difficult to acquire large-scale data, while annotation is labor-intensive and error-prone. In many cases, model performance is constrained more by the availability of reliable, well-annotated data than by architectural design.

One of the main challenges is the labeling process. Smart home data tend to be weakly structured and time-continuous, thus requiring fine-grained synchronization between different modalities. Generating such labels usually requires long-term observation and human validation, which makes the whole process difficult to scale. The prevalence of unlabeled data and the high cost of annotation have led to growing interest in self-supervised learning as a way to improve data efficiency [10], demonstrating that the issue is not only the amount of data, but also the availability of accurate and semantically meaningful annotations.

These problems are further exacerbated in multi-resident scenarios, where the activities of different people may be concurrent and overlapping. Moreover, it is difficult to generate stable labels even when data can be collected continuously. In [12], Dahal et al. attempted to address this problem using federated learning and showed that labeling users’ activities is challenging. On the other hand, the scale of publicly available datasets remains limited, especially for elderly populations and long-term observations [76,77]. Thus, the research community is no longer satisfied with merely collecting more data. Recent work has focused on addressing data scarcity and imbalance. For example, DiscHAR [78] proposed a novel approach for dataset construction, while [79] studied activity recognition from multi-user data. These studies suggest that data construction and model training should be considered jointly.

A fundamental limitation is the lack of privacy-preserving, shareable, and standardized data systems. Without addressing this issue, further improvements in model architectures are unlikely to translate into significant performance gains in real-world applications.

### 5.2. Privacy and Security Concerns

Smart home systems have inherent privacy and security limitations, rather than being affected only by external factors. The continuous observation of user behavior, device usage, and health conditions poses significant risks of data exposure, behavioral inference, and system manipulation. This implies that privacy preservation must be considered alongside model design and deployment strategies.

This is reflected in data processing architectures. The traditional cloud-based pipeline enhances computational performance but increases vulnerability to data transmission and storage risks. In response, there has been a shift toward edge intelligence. FeL-MAR systems are built on federated learning, where model training is performed across distributed nodes without sharing raw data [12], and anomaly detection at the network edge demonstrates that inference can be conducted locally with reduced latency and improved privacy [35]. This indicates a structural shift from centralized computing toward decentralized intelligence.

Nevertheless, privacy and security challenges are not limited to data locality. Even in decentralized environments, systems may be subject to model poisoning, adversarial attacks, traffic spoofing, or unauthorized access. The non-IID nature of data and the heterogeneity of IoT devices further increase system complexity. Approaches such as federated learning with blockchain-based trust mechanisms [80] or server-side parameter alignment strategies [81] illustrate how these challenges can be addressed within unified frameworks.

Another important dimension is transparency. Security and anomaly detection systems often require explainability, yet their decisions can be difficult to interpret, potentially undermining user trust. Interpretable intrusion detection systems (e.g., LSTM-based models) [48] demonstrate that it is important not only to detect threats but also to understand the underlying reasons for detection. In this way, privacy and security issues extend beyond technical challenges to encompass system interpretability and user trust.

### 5.3. Generalization and Transferability

Generalization is still an issue that has not been overcome when using deep learning models in real smart home settings. Even though models tend to perform well on public datasets or within a single household, their performance often degrades when applied to new users, spatial arrangements, or device setups. This limitation is less due to a lack of model capacity than to the high context dependency and intrinsic heterogeneity of smart home data. Sensor placement, behavioral routines, interaction styles, and device usage patterns all contribute to significant distribution shifts, which undermine the stability of models trained under controlled conditions.

User variability is one of the core issues in this challenge. Variations in motion, body shape, and interaction practices among individuals performing similar activities lead to systematic differences in sensed signals. This has been demonstrated by Ye et al., who showed that cross-user differences can be large enough to create discrepancies between training and deployment data distributions, which should be addressed through domain adaptation approaches [82]. This suggests that generalization limitations in smart homes are rooted in human behavioral diversity rather than solely in data scale. Resident-specific feature extraction through multimodal fusion may provide a complementary way to mitigate cross-user variability by combining device-free motion sensing, such as WiFi CSI or radar, with identity-sensitive wearable or physiological signals.

These problems are exacerbated by environmental variability. Changes in room layout, sensor configuration, and functional area division alter the structure of perceptual data, preventing straightforward model transfer across environments. Cross-environment domain adaptation approaches [83,84] demonstrate that such distribution shifts must be explicitly addressed rather than assuming identical data distributions. In practice, models must be robust to simultaneous changes in both user behavior and environmental conditions.

In energy management, a similar situation is observed, as models are often trained on household-specific consumption patterns and device configurations, limiting their portability. Methods such as clustering, transfer learning, and federated learning have been proposed to enable knowledge sharing across households while preserving local adaptation [85,86]. These efforts demonstrate that transferability is not solely an algorithmic issue but is fundamentally related to data heterogeneity and distribution imbalance.

Recent work has shifted from optimizing performance within a single domain toward modeling distribution mismatch. Approaches aimed at improving robustness across users, environments, and system configurations—such as domain alignment, knowledge transfer, and adaptive parameterization within graph-based domain adaptation frameworks [87]—indicate a broader trend toward robustness-oriented learning paradigms.

More broadly, generalization challenges in smart homes arise from the interaction of user variability, environmental heterogeneity, and variations in household operation patterns. Addressing these challenges requires moving beyond fixed benchmarking toward models that support dynamic and context-sensitive adaptation, balancing transferable representations with the need for individualized adjustment.

### 5.4. Edge Deployment and Real-Time Constraints

Edge deployment remains a key obstacle for practical deep learning applications in smart home systems. Unlike cloud environments with abundant computational resources, home devices are severely constrained in computation, memory, energy, and communication bandwidth. Meanwhile, many applications such as anomaly detection, voice interaction, activity recognition, and device orchestration demand prompt or near real-time responses. Such resource-constrained and latency-critical scenarios create a persistent gap between offline model performance and practical applicability.

This issue lies at the core of a mismatch between model complexity and hardware capability. High-performance deep learning models are typically associated with large numbers of parameters and high computational cost, which edge devices cannot support. According to the literature on lightweight deployment, many models with strong accuracy are found to be too slow, memory-intensive, or energy-consuming when deployed on microcontrollers or embedded systems [88]. This has prompted increased focus on lightweight model design, where efficiency is considered as important as accuracy.

Recent implementations [16,17] further illustrate this transition from high-capacity offline models to lightweight edge-oriented HAR systems. For example, lightweight multimodal fusion and spatiotemporal learning frameworks have been designed to support human action recognition directly on edge devices, reducing the dependence on cloud-based inference. IoT-oriented hybrid HAR models have also combined feature preprocessing, CNN-based representation learning, and recurrent temporal modeling to balance recognition performance with deployability. These examples reinforce that edge deployment is not limited to model compression alone, but also requires coordinated optimization of sensing modality, feature representation, temporal modeling, inference latency, and hardware resource consumption.

The challenge is further influenced by the heterogeneity of task requirements. Applications requiring instant response, such as voice interaction and anomaly detection, benefit from local inference but impose stricter constraints on latency and resource usage. Real-time operation with limited data transmission can be achieved through lightweight implementations, such as TinyML-based keyword spotting and edge-level anomaly detection systems [35,38]. Nevertheless, these approaches also reveal trade-offs between accuracy, responsiveness, and resource consumption, suggesting that deployability should be evaluated across multiple dimensions rather than a single metric.

These limitations are already influencing methodological development—recent work focuses on reducing model complexity through architectural optimization [51], parameter compression [78], and efficient network design [54], for example, in lightweight activity recognition models [89]. On the other hand, studies on deploying Transformer-based models show that even simplified architectures remain difficult to run on low-power devices [56], highlighting the limitations of directly transferring high-capacity models to edge settings. In many practical cases, feasibility under resource constraints takes precedence over marginal improvements in predictive performance.

This shift affects not only models but also system design. Instead of relying solely on cloud-based pipelines, smart home systems are increasingly designed as hierarchical architectures, where sensors and edge devices perform local computation [90]. Such structures enable low-latency local responses while still supporting more complex analysis at higher levels, indicating a shift toward system-level optimization.

In this context, edge deployment cannot be reduced to model compression alone. The challenge lies in balancing predictive performance with inference speed, energy consumption, and system complexity as an integrated whole. Without such a balance, gains achieved in offline evaluation are unlikely to translate into effective and scalable real-world deployment.

### 5.5. Explainability and User Trust

Restricted explainability and the subsequent lack of user trust are key obstacles to the long-term implementation of deep learning in smart home systems. Model outputs in smart homes, unlike traditional offline tasks, tend to trigger downstream effects such as device coordination, anomaly alerts, and automatic control. These decisions directly affect user experience, perceived safety, and willingness to adopt the system. When system behavior deviates from user expectations, the central concern is not only whether the decision is correct but whether it can be explained. Deep learning models that lack interpretable reasoning may easily erode user acceptance.

This issue is especially apparent when perception outputs are closely tied to system actions. In activity recognition and intelligent control, discrepancies between predicted states and user perception can lead to doubts about system behavior. Empirical studies suggest that users prefer natural language explanations over abstract labels, indicating that interpretability should align with human understanding rather than internal model representations [91,92]. In this regard, explainability is not an auxiliary feature but a requirement for effective human–system interaction.

The need for explanation is further intensified in high-risk applications such as security monitoring and anomaly detection. Systems that provide detection results without explainable reasoning cannot be reliably validated, even when accuracy is high. Methods such as LIME and SHAP enhance transparency by identifying the factors influencing model decisions [93], while explainable sequence models demonstrate the value of linking detected anomalies to interpretable causes [48]. In this context, explanation supports not only transparency but also verifiability and practical usability.

Meanwhile, the concept of explainability is evolving. Early approaches focused on feature attribution to reveal which inputs influence predictions. However, such explanations are often difficult for non-expert users to understand. Recent work has shifted toward user-centered explanations, aiming to convey model output in intuitive and context-aware forms. For example, large language models have been explored to generate natural-language explanations for activity recognition tasks [94], indicating a shift toward human-centered communication rather than model-centric interpretation.

Notably, explanation alone does not guarantee trust. While the absence of explanation limits user acceptance, poorly designed explanations may cause confusion or expose uncertainty, thereby reducing confidence. This highlights the need to consider explainability, user cognition, and interaction design together. In this sense, the problem is no longer simply how to make models interpretable, but how to design systems in which technical reasoning aligns with human expectations. Only through such alignment can smart home systems move beyond functional automation toward stable integration into daily life.

### 5.6. Future Research Directions

It is clear that the analysis above shows that deep learning in smart homes is moving toward system-wide optimization rather than task-specific optimization. Although research has reached a considerable level in individual tasks, relatively limited work has focused on developing solutions that are stable, scalable, and sustainable for real-world household deployment. Future studies are likely to focus less on incremental performance improvements and more on resolving the fundamental conflicts among data heterogeneity, system constraints, and user interaction.

One key direction is the development of unified perception systems capable of integrating heterogeneous sensing modalities. Single-modality perception is no longer sufficient, as the diversity of sensing technologies (radar, WiFi, audio, vision, and environmental sensors) continues to grow. Existing studies show that these signals can be combined in a complementary manner to achieve significant performance gains in complex scenarios [95]. Future work is expected to emphasize principle-driven multimodal fusion, including cross-modal alignment, adaptive weighting, and structural modeling for coherent integration of diverse sensory inputs.

Meanwhile, the tension between data utilization and privacy protection is driving the development of distributed intelligence. Instead of centralized data aggregation, emerging approaches combine federated learning, edge computing, and local inference to enable privacy-preserving training and deployment [35,96]. This reflects a broader shift toward decentralized architectures, where learning and inference are distributed across devices to operate under both resource and privacy constraints.

Another important direction is knowledge integration. Purely data-driven models have limitations in capturing long-term dependencies and contextual relationships in smart home environments. Incorporating structured knowledge, such as device graphs, spatial relationships, temporal constraints, and domain-specific evolution patterns, can enhance reasoning and improve generalization [62]. Recent studies in adjacent intelligent sensing and state-recognition domains also suggest that knowledge-driven learning can be combined with adversarial meta-learning to improve few-shot recognition under variable conditions. For example, Yin et al. proposed a fault-evolution-knowledge-driven adversarial meta-learning method, providing a useful methodological reference for integrating domain knowledge, distribution adaptation, and rapid generalization [97]. Beyond single-scenario models, future smart home systems may similarly learn transferable representations across multiple scenarios and adapt them efficiently to new users, devices, and environments.

More broadly, the emergence of large-scale pretrained and multimodal models is likely to drive convergence across perception, reasoning, and interaction within unified frameworks. Early applications of large language models for explanation and zero-shot learning [88], along with advances in multimodal foundation models [98], suggest a trend toward integrating diverse tasks within a single system. In smart home contexts, this implies systems that not only perform tasks but also understand and explain their behavior, improving both efficiency and usability. In smart home contexts, this implies systems that not only perform tasks but also understand and explain their behavior, improving both efficiency and usability. Recent work has also explored knowledge-driven, meta-learning approaches for modeling system state evolution under variable conditions, highlighting the potential of advanced learning frameworks to enhance adaptability and robustness [99].

At the same time, system behavior is increasingly shaped by human-centered requirements. Smart homes are inherently long-term, human-oriented systems in which usability, predictability, and controllability are as important as predictive accuracy. This highlights the importance of user understanding, interaction design, and feedback mechanisms in future development. Natural language interaction, personalized automation strategies, and human-in-the-loop control will be essential for bridging the gap between technical capability and user acceptance.

Taken together, these directions suggest a redefinition of smart home intelligence as a coordinated system-level problem rather than a collection of isolated tasks. Future progress will depend on the joint advancement of perception, decision-making, deployment, and human–AI interaction, with careful consideration of the balance among performance, privacy, adaptability, and usability in real-world environments.

## 6. Conclusions

This paper is a review article on deep learning in smart homes. The research trends, major application areas, methodological developments, and real-world challenges are discussed. The existing literature shows that deep learning has become a fundamental driver of smart home intelligence. While early work focused primarily on human activity recognition as a core task, recent studies have expanded to include applications such as health monitoring and assisted living, smart energy management, security surveillance and anomaly detection, and voice interaction. This expansion reflects both the flexibility of deep learning approaches and the continued growth of this research field.

On the one hand, deep learning represents a new methodological paradigm for smart home systems. Early approaches were mainly based on CNN and RNN/LSTM/GRU architectures for local feature extraction and temporal modeling. In recent years, more advanced models such as Transformers, graph-based learning, multimodal fusion, and deep reinforcement learning have been introduced to address more complex tasks and scenarios. This evolution reflects a shift from improving performance in individual tasks to enabling integrated systems that combine perception, cognition, reasoning, and decision-making.

At the same time, several challenges remain unresolved. Real-world data collection and annotation in residential environments are difficult, resulting in datasets that lack sufficient scale, diversity, and representativeness, which in turn affects model training and cross-domain generalization. The sensitive nature of smart home data also raises critical concerns regarding privacy and security, which must be addressed throughout data acquisition, model training, and deployment. Furthermore, variations in home layouts, sensor configurations, and user behavior introduce additional complexity for model transferability and system robustness. Issues related to edge deployment, model explainability, and user trust further limit large-scale and long-term adoption.

Looking ahead, future research should focus not only on performance optimization but also on system sustainability. On the technical side, directions such as multimodal collaborative perception, privacy-aware edge intelligence, knowledge-enhanced modeling, and cross-domain generalization will be essential for real-world deployment. At the same time, user-centered considerations—including explainability, interactive feedback, and human-in-the-loop mechanisms—will play a critical role in ensuring practical adoption. As long-term human-centric environments, smart homes must be designed not only for accuracy but also for stability, interpretability, and sustainability over time.

In conclusion, deep learning has provided essential methodological support for smart home research and enabled new opportunities for intelligent perception and autonomous services. Future advancements in sensing, modeling, decision-making, deployment, and human–AI interaction will be necessary to develop smart home systems that are not only intelligent but also robust, trustworthy, and sustainable for real-world adoption.

## Figures and Tables

**Table 1:** Overview of major application domains, representative tasks, and key challenges of deep learning in smart homes.

Application Domain	Main Tasks	Typical Input Data	Primary Objectives	Key Challenges
Human Activity Recognition	Activity recognition, multi-resident analysis, anomaly detection	Ambient sensors, wearables, WiFi/CSI, radar	Activity understanding and context-aware services	Annotation cost, resident interference, cross-user variability, limited generalization, privacy
Health Monitoring and Assisted Living	Fall detection, sleep monitoring, elderly care, chronic disease support	Wearable signals, motion sensors, radar, thermal data	Health risk detection and assisted living support	Rare abnormal data, individual variability, limited long-term labels, privacy
Smart Energy Management	Load forecasting, demand response, energy scheduling	Energy records, smart meter data, environmental data, electricity prices, occupancy data	Energy-efficient and cost-aware control	Weak transferability, comfort modeling, real-time constraints, distributed integration
Security Monitoring and Anomaly Detection	Intrusion detection, abnormal event detection, device anomaly monitoring	Video, network traffic, sensor logs, device telemetry	Real-time safety monitoring and anomaly detection	False alarms, privacy risks, device heterogeneity, limited interpretability
Voice Interaction and Intelligent Control	Keyword spotting, speech recognition, intent understanding, multimodal control	Speech, text, user context, gaze/gesture, radar gestures	Natural interaction and proactive control	Noise sensitivity, intent ambiguity, interoperability, control balance

**Table 2:** Functional roles of representative deep learning paradigms in smart home systems.

Methodological Paradigm/Functional Role	Core Strengths	Typical Applications	Representative Inputs	Main Limitations	Deployment Considerations
CNN-based local representation learning	Local feature extraction, parameter sharing, robustness to signal variation	Activity recognition, energy prediction, gesture recognition, visual monitoring	Sensor images, time-series segments, spectrograms, thermal/video data	Limited ability to model long-range temporal dependencies and global context	Efficient and widely deployable; often used as a front-end encoder with temporal or attention modules
RNN/LSTM/GRU-based temporal modeling	Sequential dependency modeling, temporal pattern learning	Activity recognition, anomaly detection, health monitoring, energy forecasting	Sequential sensor streams, physiological signals, energy consumption sequences, traffic sequences	Limited parallel efficiency and relatively weak long-range context modeling	Moderate computational cost; suitable for edge-based time-series analysis
Transformer-based global context modeling	Global dependency modeling, long-range context capture, flexible sequence encoding	Complex activity recognition, command understanding, energy forecasting, context-aware interaction	Long sensor sequences, WiFi CSI, multimodal signals, text or speech-command representations	High computational and memory demand; often requires large-scale data	Powerful for complex sequence and multimodal tasks, but challenging for resource-constrained devices
Graph-based relational reasoning	Structural relationship modeling, sensor-device-user interaction modeling, relational reasoning	Activity recognition, human-object interaction, context reasoning, device recommendation	Sensor graphs, device relations, interaction graphs, spatiotemporal structures	Strong dependence on graph construction quality and updating mechanisms	Useful in structured environments but may increase system complexity
Multimodal fusion strategy	Cross-modal integration, improved robustness, complementary information modeling	Health monitoring, security analysis, intent understanding, contactless recognition	Radar, WiFi, audio, video, text, environmental sensors, wearable data	Synchronization issues, missing modalities, heterogeneous data quality, high complexity	Effective in complex scenarios but often requires careful system-level design and resource management
Deep reinforcement learning for decision-making and control	Dynamic decision-making, long-term optimization, adaptive control	Energy scheduling, occupant-aware control, HVAC management, home automation	Energy states, device states, occupancy context, environmental feedback, price signals	Sample inefficiency, reward design difficulty, training instability, limited interpretability	Better suited for high-level control, especially when combined with simulation, prediction models, or edge-cloud architectures
Federated learning for distributed privacy-preserving training	Collaborative model training without sharing raw data; privacy preservation; cross-household knowledge sharing	Activity recognition, health monitoring, energy prediction, intrusion detection, edge intelligence	Local sensor streams, physiological data, energy records, network traffic, device logs	Non-IID data, communication cost, client heterogeneity, model poisoning, privacy leakage from updates	Suitable for privacy-sensitive smart home applications; requires robust aggregation, personalization, and efficient edge communication

**Table 3:** Representative public datasets commonly used in deep learning-based smart home research.

Dataset	Main Task(s)	Data Modality	Typical Application Scenarios	Main Characteristics	Reference
CASAS	Human activity recognition, anomaly detection, assisted living	Ambient sensor event streams	Long-term in-home behavior analysis, sequential behavior modeling	A benchmark ambient-sensing smart home dataset, suitable for environment-aware HAR	[1]
UCI HAR	Basic activity recognition	Smartphone inertial signals	Benchmark comparison for wearable activity recognition	A standardized inertial sensing dataset, suitable for sequence model evaluation	[4]
Toyota Smarthome	Daily activity recognition, human–object interaction analysis	RGB-D videos	Vision-based in-home activity recognition	Collected in real home environments with relatively high activity complexity	[66]
UP-Fall	Fall detection, activity recognition	Multimodal signals including video, inertial, and physiological data	Multimodal assisted care research	One of the commonly used public benchmarks for fall detection	[67]
eHomeSeniors	Privacy-preserving fall detection	Thermal infrared images	Privacy-aware elderly in-home monitoring	Based on thermal imaging, suitable for privacy-sensitive scenarios	[68]
REDD	Energy analysis, load disaggregation	Household electricity monitoring data	Non-intrusive load monitoring, household energy modeling	Provides whole-home and circuit-level electricity data	[69]
UK-DALE	Household load forecasting, load disaggregation	Whole-home and appliance-level electricity data	Appliance-level energy analysis, household electricity forecasting	Multi-household, multi-scale energy consumption dataset	[70]
REFIT	Long-term energy forecasting, occupancy behavior analysis	Smart meter and appliance-level measurement data	Long-term residential energy analysis	Multi-household dataset with long time-series records	[71]
TON_IoT	Intrusion detection, anomaly detection	Network traffic, system telemetry	IoT and smart home security analysis	Multi-source security dataset, suitable for detection model development	[72]
CICIoT2023	IoT attack detection	Network traffic	Large-scale IoT security detection	Rich attack categories, suitable for evaluating deep intrusion detection models	[73]
Speech Commands	Keyword spotting, speech command recognition	Audio speech signals	Home voice assistants, edge keyword spotting	A standard benchmark for small-vocabulary speech recognition	[74]
Fluent Speech Commands	Spoken intent understanding	Speech + intent labels	Semantic understanding of smart home commands	Emphasizes mapping spoken commands to intents and slots	[75]

**Table 4:** Common evaluation metrics across representative smart home tasks.

Task Category	Common Evaluation Metrics	What They Reflect	Reference
Human activity recognition/gesture recognition	Accuracy, Precision, Recall, F1-score	Classification performance and recognition balance	[10,11,13,15]
Health event detection/fall detection	Accuracy, Recall, Specificity, Precision	Safety-critical event detection capability	[22,24]
Anomaly detection/intrusion monitoring	Accuracy, Precision, Recall, F1-score	Abnormal event discrimination and monitoring reliability	[33,34,35]
Load forecasting	MAE, RMSE, MAPE	Error magnitude and forecasting stability	[28]
Smart home scheduling/DRL energy management	Electricity cost, user comfort, thermal comfort, revenue	Long-term control effectiveness and operational benefit	[27,63,64,65]
Edge deployment/TinyML	Inference latency, RAM/memory footprint, flash/storage footprint, parameter count, FLOPs	Deployment feasibility and resource efficiency	[38,39,46]

## Data Availability

No new data were created or analyzed in this study. Data sharing is not applicable to this article.
